# Risk of Thromboembolic Events and Major Adverse Cardiovascular Events Following Antivascular Endothelial Growth Factor Therapy in Patients with Colorectal Cancer

**DOI:** 10.3390/cancers15010009

**Published:** 2022-12-20

**Authors:** Yen-Cheng Chen, Liang-Tsai Yeh, Shun-Fa Yang, Ming-Chih Chou, Jing-Yang Huang, Chao-Bin Yeh

**Affiliations:** 1 Institute of Medicine, Chung Shan Medical University, Taichung 402, Taiwan; 2 Department of Surgery, Chung Shan Medical University Hospital, Taichung 402, Taiwan; 3 Department of Anesthesiology, Changhua Christian Hospital, Changhua 500, Taiwan; 4 School of Medicine, Chung Shan Medical University, Taichung 402, Taiwan; 5 Department of Post-Baccalaureate Medicine, College of Medicine, National Chung Hsing University, Taichung 402, Taiwan; 6 Department of Medical Research, Chung Shan Medical University Hospital, Taichung 402, Taiwan; 7 Department of Emergency Medicine, School of Medicine, Chung Shan Medical University, Taichung 402, Taiwan; 8 Department of Emergency Medicine, Chung Shan Medical University Hospital, Taichung 402, Taiwan

**Keywords:** antivascular endothelial growth factor therapy, colorectal cancer, major cardiovascular events

## Abstract

**Simple Summary:**

We assessed whether anti-VEGF therapy increases the risk of thromboembolic events or major adverse cardiovascular events (MACEs) in patients with colorectal cancer based on real-world evidence. Patients with advanced colorectal cancer who had previously received anti-VEGF therapy did not increase the risk of thromboembolic events and major cardiovascular events more than patients without anti-VEGF therapy in Taiwan.

**Abstract:**

Antivascular endothelial growth factor (anti-VEGF) therapy has been a standard treatment for patients with metastatic colorectal cancer. However, the risk of thromboembolic events and cardiovascular events associated with this therapy remains controversial. We assessed whether anti-VEGF therapy increases the risk of thromboembolic events or major adverse cardiovascular events (MACEs) in patients with colorectal cancer based on real-world evidence. This retrospective cohort study was designed using linked 2009–2016 nationwide databases, including the Taiwan Cancer Registry, the National Health Insurance Research Database, and Taiwan’s National Death Index. In total, 189,708 patients newly diagnosed as having advanced colorectal cancer from 2009 to 2016 were identified and categorized into the anti-VEGF and comparator groups through age, sex, clinical stage, and diagnosis date (within 180 days) matching. Propensity score matching was further performed to balance the baseline characteristics between the two groups. The Kaplan–Meier method was used to create the cumulative incidence curves of thromboembolic events and MACEs, and log-rank tests were used to compare the differences in Kaplan–Meier curves. Competing hazard ratios (HRs) for thromboembolic events and MACEs were estimated using the Fine–Gray method when considering the competing event of death. Statistical analysis was performed using two-tailed tests with a significance level of 0.05. In total, 4635 patients were included in both the anti-VEGF group and comparator group. The risk of thromboembolic events and MACEs did not differ significantly between the two groups. After propensity score matching, the adjusted HR for MACEs or thromboembolic events was 1.040, which for MACEs was 0.989, and that for thromboembolic events was 1.028. The competing HR for MACEs or thromboembolic events was 0.921, which for MACEs, was 0.862, and that for thromboembolic events was 0.908. In conclusion, patients with advanced colorectal cancer who received anti-VEGF therapy did not exhibit significantly higher risks of thromboembolic events and MACEs than those without anti-VEGF therapy. Our study provides real-world evidence regarding the safety of anti-VEGF therapy in Asian patients with advanced colorectal cancer.

## 1. Introduction

Colorectal cancer is the second leading cause of cancer-related deaths and the third most common cancer worldwide [1]. The incidence of colorectal cancer has been increasing worldwide, and more than 2.2 million new patients and 1.1 million colorectal cancer-related deaths have been predicted by 2030 [2]. According to the Ministry of Health and Welfare in Taiwan, colorectal cancer was the predominantly diagnosed cancer in Taiwan for 13 consecutive years until 2018. The age-standardized incidence rate (ASIR) of colorectal cancer has increased over the years, reaching 41.8 per 100,000 people in 2018. In 2018, the ASIR of colorectal cancer was 51.2 per 100,000 men and 33.6 per 100,000 women. Furthermore, almost half of the patients had advanced colorectal cancer at their initial diagnosis (24.9% in stage III and 20% in stage IV). The age-standardized mortality rate (ASMR) has also increased. Compared with 1971–1975, the ASMR of colorectal cancer increased twofold among men and 1.5-fold among women during 2006–2010 [3]. This trend is attributed not only to a Western lifestyle, genetic factors, or environmental factors, but also to the universalization of cancer screening tests and the development of the colonoscopy.

Since the 2010s, the treatment of colorectal cancer in Taiwan generally adheres to the National Comprehensive Cancer Network guidelines. If the malignancy is surgically resectable, colectomy with standard lymphadenectomy is the primary treatment. For stage II pathological colorectal cancer, uracil–tegafur, capecitabine, and fluorouracil are adjuvant treatment options [4,5]. An oxaliplatin-based regimen, such as FOLFOX and CAPEOX, is indicated for high-risk stage II and stage III pathological colorectal cancers [4,5,6,7,8,9]. For stage IV colorectal cancer, if a metachronous metastatic tumor is resectable, operation and adjuvant chemotherapy are considered. The treatment consists of an irinotecan- or oxaliplatin-based regimens, such as FOLFIRI, FOLFOX, CAPEOX, and FOLFOXIRI, with or without target therapy with vascular endothelial growth factor (VEGF) inhibitors and epidermal growth factor receptor (EGFR) inhibitors.

Angiogenesis is an essential process for tumor growth, survival, and metastasis. Hence, angiogenesis inhibition is effective in halting tumor progression. The VEGF/VEGF receptor (VEGFR) axis is one of the most crucial pathways for angiogenesis inhibition [10]. The VEGF signal can be affected by direct ligand blockade or by the inhibition of tyrosine kinases that regulate the VEGFR [11]. Bevacizumab (Avastin) uses the former mode of action, whereas regorafenib (Stivarga) uses the latter. Both these drugs are predominantly used in anti-VEGF therapy against advanced colorectal cancer in Taiwan. The benefits of anti-VEGF therapy in patients with metastatic colorectal cancer have been reported by several trials and studies, with the median overall survival increasing from 1.4 to 4.7 months and progression-free survival increasing from 1.4 to 4.5 months [12,13,14,15,16].

When assessing the mechanism of action of anti-VEGF therapy, cardiovascular toxicity must be considered. Recent studies have reported increased risks of hypertension, arterial thromboembolism, cardiac ischemia, and cardiac dysfunction following anti-VEGF therapy [17,18,19,20]. However, the association between cardiovascular toxicity and anti-VEGF therapy in patients with colorectal cancer remains unclear. This retrospective cohort study assessed the risk of thromboembolic events and major adverse cardiovascular events (MACEs) associated with anti-VEGF therapy in patients with advanced colorectal cancer treated with standard treatment with or without anti-VEGF therapy in Taiwan. We hypothesized that patients with advanced colorectal cancer who received anti-VEGF therapy would have a higher risk of thromboembolic events and MACEs.

## 2. Materials and Methods

### 2.1. Data Source

This retrospective cohort study was designed using linked nationwide databases, including the Taiwan Cancer Registry (TCR), the National Health Insurance Research Database (NHIRD), and Taiwan’s National Death Index; data from 2009 to 2016 were included. The research data sets were maintained for academic research by Taiwan’s Health and Welfare Data Science Center [21].

Since 2002, the TCR has been collecting data from hospitals by using a long-form system; the registry includes detailed information on cancer diagnosis, treatment, and prognosis. Initially, it included cancers related to the oral cavity and pharynx, colon and rectum, liver, lung, breast, cervix, and uterus. In 2009, this list was extended to include esophageal, stomach, prostate, and bladder cancers. The NHIRD consists of the following information: (1) demographics and enrolment status; (2) pharmacy dispensing (outpatient and inpatient); (3) diagnosis (ambulatory, emergency, and inpatient care), coded according to the International Classification of Diseases, Ninth Revision, Clinical Modification (ICD-9-CM) until the year 2015; (4) procedures (radiology, endoscopy, surgery, and special examinations), coded according to the local system; (5) dental care; (6) selected traditional Chinese medicine consultation and medication. Information on the survival status of the patients was obtained from the National Death Index database.

All residents in Taiwan have a unique personal identification number, which helps link information across these nationwide databases. The Institutional Review Board of the Chung Shan Medical University Hospital approved this study (IRB number: CS2-20194). All data in this study were encrypted and remained anonymous during data analysis.

### 2.2. Identification of Patients with Stage III/IV Colorectal Cancer

In total, 125,091 patients who were diagnosed with colorectal cancer (ICD-9 codes 153–154, ICD-10 codes C18–C19) from 2009 to 2016 were identified from the TCR. The following cases were excluded: missing demographic data (*n* = 325), missing information on cancer stage (*n* = 36,274), and diagnosis when deceased (*n* = 4). Since 2011, patients with stage IV colorectal cancer are eligible to receive standard chemotherapy with bevacizumab as the first-line treatment in the Taiwan National Health Insurance program. Since 2015, patients with stage IV colorectal cancer who experience disease progression after undergoing chemotherapy and target therapy are eligible to receive regorafenib as the third- or fourth-line treatment. Therefore, 52,719 patients diagnosed as having early-stage (in situ, stage I, or stage II) colorectal cancer were excluded. Finally, 35,769 patients who were diagnosed as having stage III/IV colorectal cancer from 2009 to 2016 were included in the analysis.

### 2.3. Anti-VEGF Group and Matched Comparator Group

Among the patients with advanced colorectal cancer, 8295 patients had received anti-VEGF therapy with bevacizumab (Anatomical Therapeutic Chemical [ATC] code L01XC07) and regorafenib (ATC code L01XE21 or L01EX05). In half of these patients, anti-VEGF therapy was initiated 97 days after diagnosis. Of the patients who received anti-VEGF therapy, 1029 (12.4%) patients who did not initialize anti-VEGF therapy within 2 years after cancer diagnosis were excluded due to interference from other complications and environmental factors. The date of the first anti-VEGF therapy prescription was defined as the index date. A total of 1492 anti-VEGF users who experienced thromboembolic events or MACEs before the index date and 1139 anti-VEGF users who could not be paired with a suitable comparator were excluded. Finally, 4635 patients were treated with anti-VEGF therapy. Time distribution matching was performed to deal with immortal time bias [22]. The participants in both groups were matched by age, sex, clinical stage, and diagnosis date (within 90 days) for the analysis, and they were all at risk on the index date (the index date of the comparator was the same as that of the paired anti-VEGF user patient).

However, in the observational study, the treatment was not allocated at random. The potential confounding effect may be due to the difference in baseline characteristics between the study groups. Propensity score matching was performed to balance the baseline characteristics between the study groups. To balance the baseline characteristics and clinical conditions of cancer diagnosis, the average effect of anti-VEGF therapy was estimated, and propensity score-matched comparators were then selected by using the greedy nearest neighbor matching algorithm and nonreplacement paired within 0.01 caliper width. For propensity score matching, the following covariates were considered: demographics (sex and age at the index date), cancer diagnosis (year of diagnosis and clinical stage), cancer treatment (surgery, radiotherapy, and chemotherapy), body mass index (BMI), smoking status, and comorbidities (hypertension, diabetes mellitus, depression, renal disease, anemia, autoimmune disease, chronic obstructive pulmonary disease, and peptic ulcer disease). After propensity score matching, 3350 paired anti-VEGF users and comparators were selected for propensity score analysis.

### 2.4. MACEs and Thromboembolic Events

The thromboembolic events that necessitated hospitalization included ischemic heart disease (ICD-9 codes 410–414 or ICD-10 codes I20–I25), ischemic stroke (ICD-9 codes 433–435 or ICD-10 codes I63–I65), deep vein thrombosis (ICD-9 codes 451.1–451.2, or ICD-10 codes I80.1–I80.2), and pulmonary embolism (PE, ICD-9 code 415.1 or ICD-10 codes I26.0 and I26.9). MACEs included ischemic heart disease, ischemic stroke, and heart failure (ICD-9 code 428 or ICD-10 code I50) after the index date. All participants were followed from the index date until the occurrence of thromboembolic events, MACEs, or death or until 31 December 2018.

### 2.5. Study Covariates

In this study, the covariates associated with the risk of mortality, MACEs, or thromboembolic events were as follows: demographics, including sex (male and female) and age (<40, 40–49, 50–59, 60–79, and ≥80 years) at the index date; cancer diagnosis, including year of diagnosis (2009–2012 and 2013–2016) and clinical stage (III and IV); cancer treatment (surgery, radiotherapy, and chemotherapy). BMI and smoking status were identified from the TCR data sets. BMI data of approximately 20% of the patients were missing; the BMI was classified into <18.5, 18.5–24, and >24. Moreover, data regarding smoking status were missing for approximately 20% of the patients; the smoking status was classified into never smoker, current smoker, and former smoker. The comorbidities that were defined using ICD-9 (before 2016) or ICD-10 (during or after 2016) codes were identified within 2 years before the index date from the NHIRD. These comorbidities included hypertension (ICD-9 codes 401–405, ICD-10 codes I10–I15), diabetes mellitus (ICD-9 code 250, ICD-10 codes E10–E14), chronic kidney disease (ICD-9 codes 582–586, ICD-10 codes N03, N05–N07, N16–N19, E10.21, E11.21), and hyperlipidemia (ICD-9 codes 272.0–272.3, ICD-10 codes E78.0–E78.5).

### 2.6. Statistical Analysis

Analysis using a large sample is likely to reveal a statistically significant difference at *p* < 0.05 even if the effect size is negligible or small [23]. In this large-sample observational study, the absolute standardized difference (ASD) was used to compare the statistical values of baseline covariates between the groups. The characteristics were balanced when the ASD was <0.1. The incidence of thromboembolic events or MACEs was defined as the number of events divided by the sum of person-years within the follow-up interval. The Kaplan–Meier method was used to create cumulative incidence curves. Log-rank tests were used to compare the overall cumulative rates between the anti-VEGF and comparator groups. Moreover, the multivariable Cox proportional hazard model was used to estimate the hazard ratio (HR) and 95% confidence interval (CI). The Fine–Gray method was used to assess the competing HRs for thromboembolic events and MACEs when considering the competing event of death [24]. SAS (version 9.4; SAS Institute, Cary, NC, USA) was used for statistical analysis. The significance level was set at 0.05, and a two-tailed test was used to assess the association between anti-VEGF therapy and the risk of thromboembolic events and MACEs.

## 3. Results

### 3.1. Baseline Characteristics

**Figure 1:**
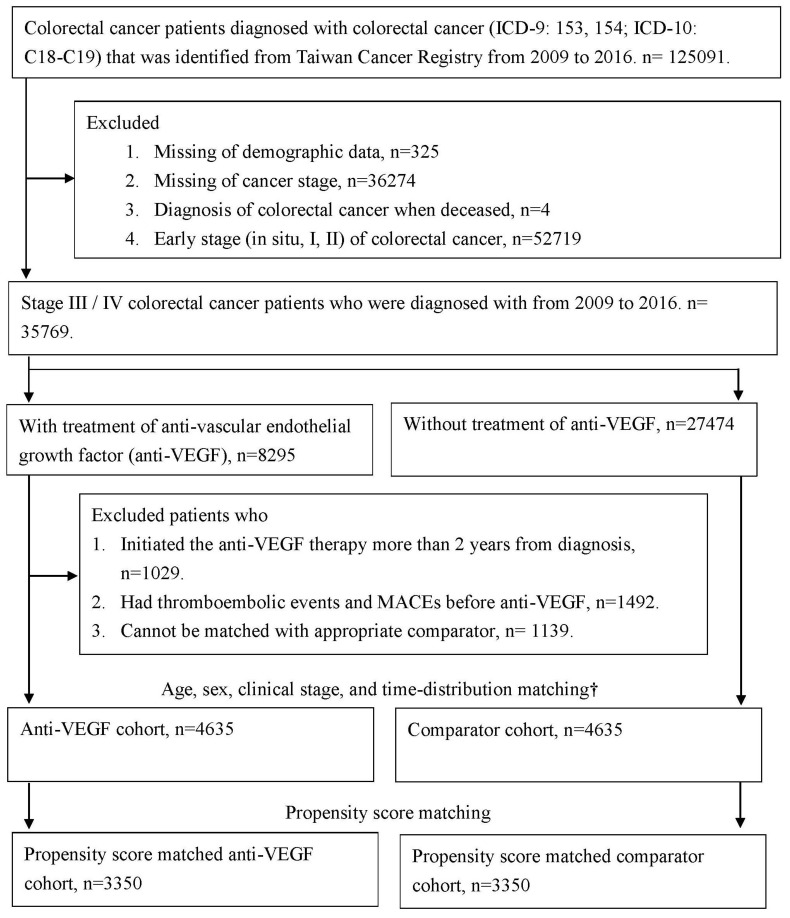
Flow chart of selected colorectal cancer patients who were treated with anti-vascular endothelial growth factor (anti-VEGF) and the comparator cohort. † Age, sex, clinical stage, and time-distribution matching was used to deal the immortal time bias, the comparators, who were matched with anti-VEGF patient by age, sex, clinical stage, and diagnosis date, and at risk on the index date (comparator’s index date was the same as the paired anti-VEGF patient). Abbreviation: MACEs, Major cardiovascular events; anti-VEGF, anti-vascular endothelial growth factor.

In this study, 52,719 patients diagnosed as having early-stage (in situ, stage I, or stage II) colorectal cancer were excluded. Finally, 35,769 patients who were diagnosed as having stage III/IV colorectal cancer from 2009 to 2016 were included in the analysis (Figure 1). The baseline characteristics are listed in Table 1. Before propensity score matching, among the age-, sex-, clinical stage-, and time distribution-matched participants, 55.8% were male, 70.2% were diagnosed as having clinical stage IV cancer, and approximately 83% were older than 50 years. ASD > 0.1000 was used to identify the unbalanced characteristics that may contribute to the confounding effect on the study results. More patients received surgical treatment (77.6% versus 73.2%) and chemotherapy (94.2% versus 68.0%) in the anti-VEGF group than in the comparator group. More patients had a baseline BMI > 24 in the anti-VEGF group (33.53%) compared with the comparator group (28.69%). Moreover, a higher proportion of never-smokers was noted in the anti-VEGF group (61.04%) than in the comparator group (55.73%). A lower prevalence of chronic kidney disease was noted in the anti-VEGF group (5.57%) than in the comparator group (9.04%) at the baseline. After propensity score matching, all the baseline characteristics were balanced (ASD < 0.1000) between the two groups.

### 3.2. MACEs, Thromboembolic Events, and Mortality Risk between the Study Groups

The risk of MACEs, thromboembolic events, and mortality are presented in Table 2. For all MACEs or thromboembolic events among age-, sex-, clinical stage-, and time distribution-matched groups, the incidence rate (95% CI) per 1000 person-months was 2.30 (2.00–2.64) in the anti-VEGF group and 2.41 (2.11–2.74) in the comparator group; the crude HR (95% CI) and adjusted HR (aHR) (95% CI) were 0.989 (0.798–1.224) and 1.040 (0.818–1.322), respectively. Regarding the risk of MACEs, the incidence rate was 2.00 (1.72–2.32) in the anti-VEGF group and 2.28 (1.99–2.60) in the comparator group; the crude HR and aHR, adjusted for age, sex, clinical stage, treatment, BMI, smoking, and comorbidities, were 0.919 (0.734–1.151) and 0.989 (0.769–1.273), respectively. Regarding the risk of thromboembolic events, the incidence rate was 1.88 (1.62–2.20) in the anti-VEGF group and 1.94 (1.68–2.24) in the comparator group; the crude HR and aHR were 0.973 (0.769–1.231) and 1.028 (0.789–1.340), respectively. The mortality rate was 138.68 (37.41–40.00) in the anti-VEGF group and 25.32 (24.36–26.32) in the comparator group; the crude HR and aHR were 1.920 (1.796–2.053) and 1.540 (1.431–1.658), respectively. After propensity score matching, the aHR (95% CI) for MACEs or thromboembolic events, MACEs, thromboembolic events, and mortality was 1.046 (0.838–1.306), 1.004 (0.796–1.267), 1.053 (0.821–1.352), and 1.637 (1.537–1.743), respectively.

**Figure 2:**
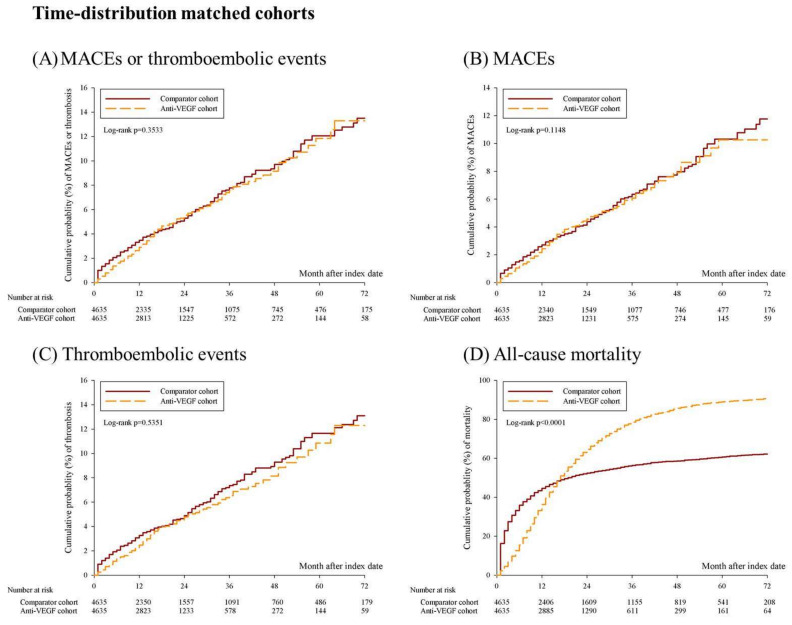

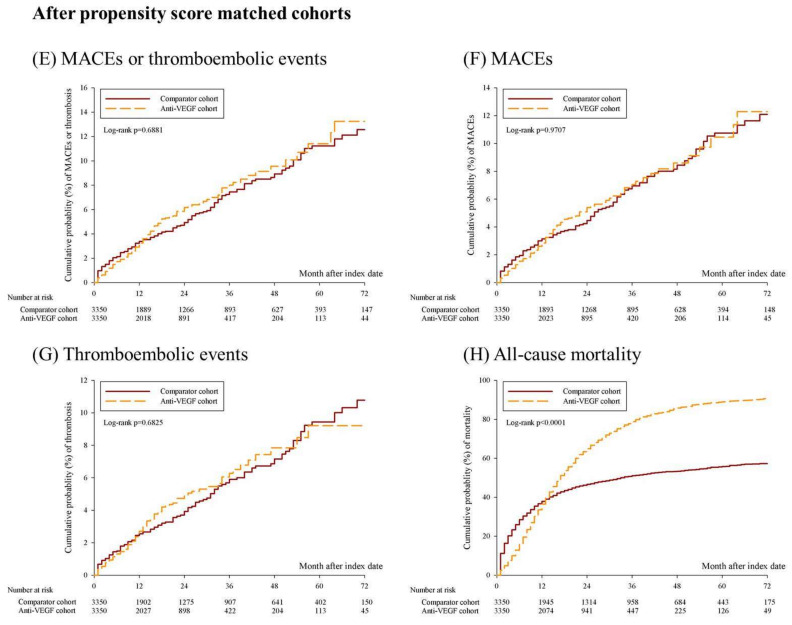
Kaplan–Meier curves for the 6-year cumulative probability of MACEs, thromboembolic events, and all-cause mortality among anti-VEGF cohort and comparator cohort. (**A**–**D**) shows the cumulative probability of MACEs or thromboembolic events, MACEs, thromboembolic events, and all-cause mortality among age, sex, clinical stage, and time-distribution matched cohorts. (**E**–**H**) shows the cumulative probability of MACEs or thromboembolic events, MACEs, thromboembolic events, and all-cause mortality among propensity score matched cohorts. Abbreviation: MACEs, major cardiovascular events.

Among the age-, sex-, clinical stage-, and time distribution-matched cohorts, the Kaplan–Meier curve revealed significantly increased mortality in the anti-VEGF group (Figure 2D, log-rank *p* < 0.0001); however, the risk of MACEs or thromboembolic events (Figure 2A, log-rank *p* = 0.3533), MACEs (Figure 2B, log-rank *p* = 0.1148), and thromboembolic events (Figure 2C, log-rank *p* = 0.5351) did not increase significantly. After propensity score matching, the Kaplan–Meier curves for mortality remained significantly different (Figure 2H, log-rank *p* < 0.0001); however, MACEs or thromboembolic events (Figure 2E, log-rank *p* = 0.6881), MACEs (Figure 2F, log-rank *p* = 0.9707), and thromboembolic events (Figure 2G, log-rank *p* = 0.6825) were not statistically significant.

### 3.3. Stratified Analysis and Competing Risk Analysis

The results of the cancer stage-stratified analysis are presented in Table 3. In the subgroup of clinical stage III patients, no significant increase was noted in the risk of MACEs or thromboembolic events; however, significantly increased mortality (aHR = 8.878, 95% CI = 7.379–10.682) was observed. In the subgroup of clinical stage IV patients, no significant increase was noted in the risk of MACEs or thromboembolic events. Similarly, the mortality risk did not increase significantly. The subevent analysis and competing HR are reported in Table 4. When the risk was stratified by the subevent of MACEs or thromboembolic events, the aHR (95% CI) for ischemic heart disease, heart failure, ischemic stroke, venous thromboembolism, and cardiac catheterization or coronary artery bypass graft was 0.920 (0.655–1.292), 0.939 (0.566–1.556), 0.655 (0.362–1.185), 1.557 (0.645–3.759), and 0.941 (0.505–1.753), respectively. When all-cause mortality was considered as the competing event, the competing aHR (95% CI) for MACEs or thromboembolic events, MACEs, and thromboembolic events was 0.921 (0.728–1.165), 0.862 (0.675–1.101), and 0.908 (0.704–1.171), respectively.

## 4. Discussion

Using the NHIRD, the TCR, and Taiwan’s National Death Index, we enrolled 189,708 patients with newly diagnosed advanced colorectal cancer from 2009 to 2016 and assessed the relationship between anti-VEGF therapy and the risk of thromboembolic events and MACEs through 1:1 age, sex-, clinical stage, and diagnosis date matching and propensity score matching. Both results indicated that patients with advanced colorectal cancer who had received anti-VEGF therapy did not exhibit higher risks of thromboembolic events and MACEs than those without anti-VEGF therapy. Moreover, the anti-VEGF group exhibited an increased risk of mortality, resulting from the stage III population.

In the past decade, similar studies have tried to assess the safety of anti-VEGF therapy for cancer treatment. Faruque et al. [18] used 11 different VEGF inhibitors, namely, axitinib, bevacizumab, sunitinib, sorafenib, vandetanib, neovastat, cediranib, pazopanib, IM 862, PTK/ZA, and motesanib, for patients with different types of cancer and reported that the use of VEGF inhibitors potentially increased the risk of important adverse effects, such as myocardial infarction, arterial thromboembolism, hypertension, and new proteinuria, in patients with cancer. Moreover, Abdel-Qadir et al. [19] used 11 different VEGF inhibitors, namely, aflibercept, axitinib, bevacizumab, cabozantinib, pazopanib, ramucirumab, regorafenib, sunitinib, sorafenib, vandetanib, and vatalanib, in patients with different types of cancer and reported a substantial increase in the risk of hypertension, cardiac ischemia, cardiac dysfunction, and arterial thromboembolism following anti-VEGF therapy. Furuya-Kanamori et al. [20] used eight VEGF inhibitors, namely, axitinib, lenvatinib, nintedanib, pazopanib, regorafenib, sunitinib, sorafenib, and vandetanib, in patients with different types of cancer; however, despite evidence supporting an increased risk of bleeding with sunitinib treatment, none of these VEGF inhibitors were associated with cardiovascular events, including thrombotic events, myocardial infarction, stroke, venous thrombosis, PE, left ventricular dysfunction, and QT prolongation. Thus, the results of previous studies analyzing the risks of cardiovascular and hematological adverse events following anti-VEGF therapy may be overestimated. In comparison, our study design focused on patients with colorectal cancer who received anti-VEGF therapy with bevacizumab and regorafenib. No significant difference was noted in the risks of thromboembolic events and MACEs in patients who received anti-VEGF therapy and those who did not.

Several cohort studies have assessed the safety of bevacizumab in patients with metastatic colorectal cancer. Studies in the United States [25], Japan [26], and Canada [27] have concluded that bevacizumab is generally well tolerated in such patients. The incidence rates of serious adverse events, such as a thromboembolic event (1.3–2%), gastrointestinal perforation (0.9–1.9%), bleeding (2.2–10.5%), and wound healing complication (0.4–4.4%), were similar to those reported in previous randomized controlled trials. However, a study conducted in Greece [28], which enrolled not only patients with metastatic colorectal cancer but also those with metastatic breast cancer, reported a higher incidence of coronary artery disease (19.23% versus 0%, *p* = 0.151), acute myocardial infarction (14.81% versus 0%, *p* = 0.238), and thromboembolic events (17.86% versus 0%, *p* = 0.171) in the bevacizumab group than in the control group. The opposite result may be attributed to a longer median follow-up time, a smaller sample size, or different patient groups.

The beneficial effects of anti-VEGF therapy on increasing median overall survival and progression-free survival in patients with metastatic colorectal cancer have been reported by several trials and studies [12,13,14,15,16]. Our study also revealed a better mortality rate in stage IV patients following anti-VEGF therapy, which is in accordance with real-world data. However, the result of increased mortality in stage III patients must be illustrated. Importantly, a report on the National Health Insurance in Taiwan stated that patients with stage IV colorectal cancer can be treated with anti-VEGFs covered by the National Health Insurance, whereas those with stage III colorectal cancer cannot. Therefore, the stage III patients in the anti-VEGF group were those who experienced cancer progression to stage IV. Thus, the higher mortality rate could be attributed to progressive disease. Nevertheless, even with the higher mortality rate, the risks of thromboembolic events and MACEs remained neutral.

The databases (LHID and TCDB) used in this study are randomly and largely sampled from the NHIRD; therefore, the results are likely to be accurate and reliable. However, several limitations of this study should be considered. First, because this is a retrospective study, misclassification bias could not be avoided. To clarify this concern, a prospective study is warranted. Second, data regarding the difference in chemotherapy regimens and regarding tumor progression were unavailable; these factors play a crucial role in cancer treatment and may directly lead to a different outcome. We attempted to overcome this limitation by matching age, sex, clinical stage, treatment, BMI, smoking status, and baseline comorbidities between the two groups. Third, neither Taiwan Cancer Registry nor the National Health Insurance Research Database can identify cancer progression, which limits us from evaluating the efficacy of anti-VEGF treatment on survival probability in patients initially diagnosed with stage III colorectal cancer. Furthermore, we tried to balance these covariates between the two groups through PSM. Finally, as this is a Chinese-based cohort study, further studies are warranted for application to other races.

## 5. Conclusions

In this study, we did not observe the increased risks of thromboembolic events and MACEs in advanced colorectal cancer patients who had received anti-VEGF therapy than those without anti-VEGF therapy. The higher mortality rate in the anti-VEGF users with an initial diagnosis of stage III colorectal cancer was attributed to cancer progression. However, even with the higher mortality rate, the risks of thromboembolic events and MACEs were not significantly different between the two groups.

## Figures and Tables

**Table 1:** The baseline characteristics among time-distribution matched and propensity score matched study groups.

Variables	Age, Sex, Clinical Stage, and Time-Distribution Matched Groups			Propensity Score Matched Groups		
	Comparator *n* = 4635	Anti-VEGF *n* = 4635	ASD	Comparator *n* = 3350	Anti-VEGF *n* = 3350	ASD
Year of diagnosis			0.0289			0.0037
2009–2012	1778 (38.36%)	1713 (36.96%)		1344 (40.12%)	1338 (39.94%)	
2013–2016	2857 (61.64%)	2922 (63.04%)		2006 (59.88%)	2012 (60.06%)	
Sex			0.0000			0.0156
Male	2588 (55.84%)	2588 (55.84%)		1873 (55.91%)	1899 (56.69%)	
Female	2047 (44.16%)	2047 (44.16%)		1477 (44.09%)	1451 (43.31%)	
Age			0.0595			0.0697
<40	246 (5.31%)	264 (5.70%)		195 (5.82%)	176 (5.25%)	
40–49	490 (10.57%)	514 (11.09%)		387 (11.55%)	372 (11.10%)	
50–59	1157 (24.96%)	1146 (24.72%)		868 (25.91%)	845 (25.22%)	
60–79	1331 (28.72%)	1391 (30.01%)		968 (28.90%)	994 (29.67%)	
≥80	1411 (30.44%)	1320 (28.48%)		932 (27.82%)	963 (28.75%)	
Clinical Stage			0.0000			0.0697
III	1381 (29.80%)	1381 (29.80%)		1094 (32.66%)	986 (29.43%)	
IV	3254 (70.20%)	3254 (70.20%)		2256 (67.34%)	2364 (70.57%)	
Surgery	3392 (73.18%)	3596 (77.58%)	0.1023	2626 (78.39%)	2549 (76.09%)	0.0548
Radiotherapy	265 (5.72%)	314 (6.77%)	0.0437	224 (6.69%)	253 (7.55%)	0.0337
Chemotherapy	3152 (68.00%)	4367 (94.22%)	0.7107	3082 (92.00%)	3083 (92.03%)	0.0011
BMI			0.2110			0.0423
Missing	1139 (24.57%)	894 (19.29%)		786 (23.46%)	787 (23.49%)	
<18.5	368 (7.94%)	251 (5.42%)		205 (6.12%)	221 (6.60%)	
18.5–24	1798 (38.79%)	1936 (41.77%)		1328 (39.64%)	1308 (39.04%)	
>24	1330 (28.69%)	1554 (33.53%)		1031 (30.78%)	1034 (30.87%)	
Smoking			0.1551			0.0503
Missing	1007 (21.73%)	762 (16.44%)		696 (20.78%)	680 (20.30%)	
Never smoker	2583 (55.73%)	2829 (61.04%)		1882 (56.18%)	1910 (57.01%)	
Current smoker	663 (14.30%)	687 (14.82%)		499 (14.90%)	473 (14.12%)	
Former smoker	382 (8.24%)	357 (7.70%)		273 (8.15%)	287 (8.57%)	
Co-morbidity						
Hypertension	1807 (38.99%)	1841 (39.72%)	0.0150	1257 (37.52%)	1345 (40.15%)	0.0539
Diabetes mellitus	1023 (22.07%)	999 (21.55%)	0.0125	693 (20.69%)	774 (23.10%)	0.0585
Hyperlipidemia	776 (16.74%)	815 (17.58%)	0.0223	549 (16.39%)	609 (18.18%)	0.0474
Chronic kidney disease	419 (9.04%)	258 (5.57%)	0.1338	230 (6.87%)	222 (6.63%)	0.0095

ASD, absolute standardized difference. An absolute standardized difference above 0.10 is typically used as a reference point to indicate a meaningful imbalance of covariate between study groups.

**Table 2:** The risk of MACEs, thromboembolic events, and all-cause mortality among time-distribution matched groups and propensity score matched groups.

Variables	Age, Sex, Clinical Stage, and Time-Distribution Matching			Propensity Score Matched Matching		
	Comparator *n* = 4635	Anti-VEGF *n* = 4635	*p* Value	Comparator *n* = 3350	Anti-VEGF *n* = 3350	*p* Value
**MACEs or thromboembolic events**						
Observed person-months	96,014	85,664		77,897	62,033	
Newly diagnosed event	231	197		172	154	
Incidence rate (95% C.I.)	2.41 (2.11–2.74)	2.30 (2.00–2.64)	0.6415	2.21 (1.90–2.56)	2.48 (2.12–2.91)	0.2909
Crude HR (95% C.I.)	Reference	0.989 (0.798–1.224)	0.9162			
aHR (95% C.I.)	Reference	1.040 (0.818–1.322)	0.7476	Reference	1.046 (0.838–1.306)	0.6881
**MACEs**						
Observed person-months	96,164	85,956		78,042	62,244	
Newly diagnosed event	219	172		161	137	
Incidence rate (95% C.I.)	2.28 (1.99–2.60)	2.00 (1.72–2.32)	0.2042	2.06 (1.77–2.41)	2.20 (1.86–2.60)	0.5774
Crude HR (95% C.I.)	Reference	0.919 (0.734–1.151)	0.4622			
aHR (95% C.I.)	Reference	0.989 (0.769–1.273)	0.9326	Reference	1.004 (0.796–1.267)	0.9708
**Thromboembolic events**						
Observed person-months	96,944	85,981		78,712	62,319	
Newly diagnosed event	188	162		137	122	
Incidence rate (95% C.I.)	1.94 (1.68–2.24)	1.88 (1.62–2.20)	0.7879	1.74 (1.47–2.06)	1.96 (1.64–2.34)	0.3449
Crude HR (95% C.I.)	Reference	0.973 (0.769–1.231)	0.8168			
aHR (95% C.I.)	Reference	1.028 (0.789–1.340)	0.8364	Reference	1.053 (0.821–1.352)	0.6823
**All-cause mortality**						
Observed person-months	100,751	88,468		81,643	64,220	
Death	2551	3422		1666	2499	
Mortality rate (95% C.I.)	25.32 (24.36–26.32)	38.68 (37.41–40.00)	<0.0001	20.41 (19.45–21.41)	38.91 (37.42–40.47)	<0.0001
Crude HR (95% C.I.)	Reference	1.920 (1.796–2.053)	<0.0001			
aHR (95% C.I.)	Reference	1.540 (1.431–1.658)	<0.0001	Reference	1.637 (1.537–1.743)	<0.0001

Incidence rate, per 1000 person-months. aHR, adjusted for age, sex, clinical stage, cancer therapy, BMI, smoking, and co-morbidities. Abbreviation: MACEs, major cardiovascular events; anti-VEGF, anti-vascular endothelial growth factor; ASD, absolute standardized difference; aHR, adjusted hazard ratios; C.I., confidence interval; BMI, body mass index.

**Table 3:** The risk of MACEs, thromboembolic events, and all-cause mortality stratified by cancer stage.

Variables	In Stage III			In Stage IV		
	Comparator *n* = 1381	Anti-VEGF *n* = 1381	*p* Value	Comparator *n* = 3254	Anti-VEGF *n* = 3254	*p* Value
**MACEs or thromboembolic events**						
Observed person-months	47,698	27,095		48,316	58,569	
Newly diagnosed event	96	50		135	147	
Incidence rate (95% C.I.)	2.01 (1.65–2.46)	1.85 (1.40–2.43)	0.6188	2.79 (2.36–3.31)	2.51 (2.14–2.95)	0.3681
Crude HR (95% C.I.)	Reference	0.852 (0.595–1.219)	0.3794	Reference	1.021 (0.775–1.344)	0.8827
aHR (95% C.I.)	Reference	1.131 (0.755–1.693)	0.5500	Reference	0.945 (0.696–1.284)	0.7179
**MACEs**						
Observed person-months	47,723	27,171		48,441	58,785	
Newly diagnosed event	94	44		125	128	
Incidence rate (95% C.I.)	1.97 (1.61–2.41)	1.62 (1.21–2.18)	0.2837	2.58(2.17–3.07)	2.18 (1.83–2.59)	0.1769
Crude HR (95% C.I.)	Reference	0.783 (0.539–1.137)	0.1992	Reference	0.972 (0.726–1.302)	0.8508
aHR (95% C.I.)	Reference	1.044 (0.688–1.583)	0.8396	Reference	0.931 (0.673–1.288)	0.6657
**Thromboembolic events**						
Observed person-months	48,176	27,267		48,768	58,714	
Newly diagnosed event	83	36		105	126	
Incidence rate (95% C.I.)	1.72 (1.39–2.14)	1.32 (0.95–1.83)	0.1823	2.15(1.78–2.61)	2.15 (1.80–2.56)	0.9802
Crude HR (95% C.I.)	Reference	0.716 (0.473–1.083)	0.1138	Reference	1.074 (0.795–1.450)	0.6428
aHR (95% C.I.)	Reference	0.947 (0.596–1.505)	0.8185	Reference	1.011 (0.723–1.416)	0.9472
**All-cause mortality**						
Observed person-months	50,310	27,961		50,441	60,507	
Death	175	898		2376	2524	
Mortality rate (95% C.I.)	3.48 (3.00–4.03)	32.12 (30.08–34.29)	<0.0001	47.10 (45.25–49.04)	41.71 (40.12–43.37)	<0.0001
Crude HR (95% C.I.)	Reference	9.529 (8.015–11.329)	<0.0001	Reference	1.034 (0.960–1.115)	0.3778
aHR (95% C.I.)	Reference	8.878 (7.379–10.682)	<0.0001	Reference	0.974 (0.897–1.057)	0.5265

Incidence rate, per 1000 person-months. aHR, adjusted for age, sex, cancer therapy, BMI, smoking, and co-morbidities Abbreviation: MACEs, major cardiovascular events; anti-VEGF, anti-vascular endothelial growth factor; ASD, absolute standardized difference; aHR, adjusted hazard ratios; C.I., confidence interval; BMI, body mass index.

**Table 4:** The adjusted and competing hazard ratio of sub-events in anti-VEGF cohort compared with comparator cohort.

Variables	aHR (95% CI)	Competing HR (95% CI)
MACEs or thromboembolic events	1.040 (0.818–1.322)	0.921 (0.728–1.165)
MACEs	0.989 (0.769–1.273)	0.862 (0.675–1.101)
Thromboembolic events	1.028 (0.789–1.340)	0.908 (0.704–1.171)
Ischemic heart disease	1.051 (0.732–1.508)	0.920 (0.655–1.292)
Heart failure	1.111 (0.677–1.824)	0.939 (0.566–1.556)
Ischemic stroke	0.813 (0.426–1.551)	0.655 (0.362–1.185)
Venous thromboembolism	1.436 (0.623–3.310)	1.557 (0.645–3.759)
Pulmonary embolism	Not estimation	Not estimation
Cardiac catheterization or CABG	0.992 (0.534–1.844)	0.941 (0.505–1.753)

aHR, adjusted for age, sex, cancer therapy, BMI, smoking, and co-morbidities. The competing HRs of thromboembolic events or MACEs were estimated by Fine–Gray method when considering the competing event of death.

## Data Availability

Restrictions apply to the availability of these data. Data were obtained from National Health Insurance database and are available from the authors with the permission of National Health Insurance Administration of Taiwan.
